# A Thermal Performance Analysis and Comparison of Fiber Coils with the D-CYL Winding and QAD Winding Methods

**DOI:** 10.3390/s16060900

**Published:** 2016-06-16

**Authors:** Xuyou Li, Weiwei Ling, Kunpeng He, Zhenlong Xu, Shitong Du

**Affiliations:** College of Automation, Harbin Engineering University, Harbin 150001, Heilongjiang, China; lixuyou@hrbeu.edu.cn (X.L.); hekunpeng@hrbeu.edu.cn (K.H.); xzlsdlg@163.com (Z.X.); dust_1028@163.com (S.D.)

**Keywords:** fiber coil, double-cylinder (D-CYL), quadrupolar (QAD), finite element method (FEM), interferometric fiber optic gyroscope (IFOG)

## Abstract

The thermal performance under variable temperature conditions of fiber coils with double-cylinder (D-CYL) and quadrupolar (QAD) winding methods is comparatively analyzed. Simulation by the finite element method (FEM) is done to calculate the temperature distribution and the thermal-induced phase shift errors in the fiber coils. Simulation results reveal that D-CYL fiber coil itself has fragile performance when it experiences an axially asymmetrical temperature gradient. However, the axial fragility performance could be improved when the D-CYL coil meshes with a heat-off spool. Through further simulations we find that once the D-CYL coil is provided with an axially symmetrical temperature environment, the thermal performance of fiber coils with the D-CYL winding method is better than that with the QAD winding method under the same variable temperature conditions. This valuable discovery is verified by two experiments. The D-CYL winding method is thus promising to overcome the temperature fragility of interferometric fiber optic gyroscopes (IFOGs).

## 1. Introduction

The interferometric fiber optic gyroscope (IFOG) is an inertial instrument extensively used in navigation, orientation, and stabilization systems in recent years [1]. The principal configuration of IFOG consists of six parts: light source, optical coupler, Y-branch, electro-optical phase modulator, photoelectric detector and fiber coil. It is well know that the stability performance of an IFOG is greatly worsened under the influence of time-varying temperature gradients, especially asymmetric temperature excitation, therefore the key problem in developing highly-accurate IFOGs is the compensation of thermally induced drifts errors [2,3,4,5,6,7,8,9,10,11,12,13,14,15,16,17,18,19,20,21]. Considering the characteristics of IFOGs, we concluded that thermally induced drift errors are mainly caused by the following five factors: unstable wavelength of the light source, the temperature drift of integrated optical devices (Y waveguide) [2], unstable performance of electronics, unstable performance of PN dark current and thermal noise of the photoelectric detector, and thermally induced changes of the refractive index of the fiber coil. In order to overcome the thermally induced drift errors, some measures can be adopted to suppress the inducing factors. Firstly, to exclude the influence of the light source wavelength on spectral characteristics under temperature variation conditions, an erbium-doped superfluorescent fiber source (SFS) is adopted [3]. Secondly, the half-wave voltage parameters of Y waveguide are easily affected by temperature, which thereby influences the linearity and stability of IFOGs. One solution is to establish a second close-loop to compensate for the voltage fluctuation error [4], and another solution is to use some kind of thermal resistor [2]. Thirdly, aiming at the drift of electrical zero caused by unstable performance of the ancillary electronics under temperature variations, one solution is to use specially designed case components and heat sinks. Fourthly, varying temperature can cause the change of PN junction dark current and thermal noise, but so far there is no satisfactory method to eliminate the disturbance. Presently, all we can do is to select a high-quality photoelectric detector and increase photoelectric detector’s heat emission. Fifthly, varying temperature can cause changes not only of the thermal expansion coefficient and consequently the refraction index of the fiber, but also mutual extrusion pressure between the fiber coating and silica fiber, and this pressure change can result in the extrusion on the silica fiber and subsequently a change in the fiber refractive index [5,6,7]. 

To minimize the thermally induced drift error caused by refractive index changes, four methods can be adopted. The first method is to use some kind of fiber material with temperature-insensitive properties, such as air-core photonic bandgap fiber (PBF) [8,9]. The second method is to use a novel adhesive. In the limited temperature range, the thermal parameters of the potting adhesive (such as expansion coefficient, thermal conductivity and modulus) change with the temperature, so the stress distribution in the fiber coil will differ, which can destroy the stability. Therefore, in order to make the thermal parameters of the potting material match those of the winding optical fiber, we have to identify some novel adhesives [10]. The third method is to use a kind of temperature homogenizer [11]. The fourth method is to improve the winding quality of the fiber coil. For example, quadrupolar (QAD) [12], random [13], octupolar [14], cross [15,16], crossover-free [17] and double-cylinder (D-CYL) [18] winding methods have all been investigated to reduce thermally induced nonreciprocal drift errors. Some time ago, the thermal performance of the cross winding method and the D-CYL were briefly compared, but only by a simulation method [19]. However, the advantages and disadvantages of D-CYL and QAD fiber coil under asymmetric temperature excitation conditions and variable temperature excitation conditions have not been investigated and compared, in particular by experiments, and that is the main purpose of this study.

## 2. Theory

In the IFOG sensing coil, the two counter propagating beams do not experience the same shift under transient thermal fluctuation. It is difficult to distinguish between the nonreciprocal phase shift caused by a temperature fluctuation and the true, rotation induced Sagnac effect.

Two equivalent thermally induced shift error expressions can be expressed as Equation (1) [22] and Equation (2) [14], respectively:

(1) ΩE(t)=nDL∂n∂T∫0L2[T˙(l,t)−T˙(L−l,t)](L−2l)dl

(2) $$\Omega_{E}\left(t\right)=\frac{n}{DL}\frac{\partial n}{\partial T}\int_{0}^{L}\left(\dot{T}(l,t)\right)\left(L-2l\right)dl$$

where (*n/DL*)·(*δn*/*δT*) is the amplitude of the Shupe effect; *D* and *L* are the coil diameter and fiber length, respectively; *n* is the refractive index of fiber; *δn*/*δT* is the temperature coefficient (the quartz’s temperature coefficient is approximately 10^−^^5^/°C); *T*(*l,t*) is the temperature distribution along the fiber at time point *t*; $\dot{T}\left(l,t\right)$ and $\dot{T}\left(L-l,t\right)$ are the time partial derivatives of this distribution.

From Equation (1), it is seen that fiber sections more distant from the fiber midpoint contribute more substantially to a phase shift than closer ones. From Equations (1) and (2), it is also seen that if the fiber coil is provided with a spatially symmetrical distribution on both sides of the fiber coil midpoint, the thermally induced error can be offset. Therefore, we need to continuously explore some novel fiber winding techniques [12,13,14,15,16,17,18,19]. However, these winding techniques do still not yield the necessary accuracy. The reason is that while an ideal fiber coil may be effective to suppress the Shupe effect in a specific direction, it is not effective for every direction from which a temperature gradient can traverse the coil, so fiber coils need to be maintained with a symmetrical temperature distribution simultaneously. One solution to this problem is to use a heat-off spool. The heat-off spool not only slows down the temperature field variations of the fiber coil (*i.e.*, reducing the values of $\dot{T}\left(l,t\right)$ and $\dot{T}\left(L-l,t\right)$), but also accelerates temperature smoothing within the coil (reduction of the difference of $\dot{T}\left(l,t\right)-\dot{T}\left(L-l,t\right)$). In combination with special fiber winding techniques, these steps allow reaching the necessary accuracy.

For calculating the total thermally induced rate error $\Omega_{E}(t)$ quantitatively, it is necessary to make the coil in a discrete way. In order to enhance the efficiency, the thermal symmetry of optical fiber coils is considered. Therefore, the three-dimensional (3D) model could be simplified to two-dimensional (2D) model. The total length *L* of fiber is divided into *M* layers. Each layer contains *N* loops and total optic fiber loops is *MN*. By splitting the integral into partial ones, Equation (2) can be transformed into Equation (3).

(3) $$\begin{array}{l} \Omega_{E}\left(t\right)=\frac{n}{DL}\frac{\partial n}{\partial T}\{\int_{l_{1}}^{l_{1}+dl_{1}}\dot{T}\left(l_{1},t\right)\left(L-2l\right)dl+\int_{l_{2}}^{l_{2}+dl_{2}}\dot{T}\left(l_{2},t\right)\left(L-2l\right)dl+\cdots \cdots \\ \;+\int_{l_{\left(MN-1\right)}}^{l_{\left(MN-1\right)}+dl_{\left(MN-1\right)}}\dot{T}\left(l_{\left(MN-1\right)},t\right)\left(L-2l\right)dl+\int_{l_{MN}}^{l_{MN}+dl_{MN}}\dot{T}\left(l_{MN},t\right)\left(L-2l\right)dl\} \end{array}$$

where *l_i_*, *dl_i_*, and $\dot{T}$(*l_i_,t*) indicate the starting point coordinates, the length, and temperature change rate respectively, of *i-*th turn fiber. Taking this into account and generalizing, a numerical expression of $\Omega_{E}\left(t\right)$ is shown in Equation (4):

(4) $$\Omega_{E}\left(t\right)=\frac{n}{DL}\frac{\partial n}{\partial T}\sum_{i=1}^{MN}\left(\dot{T}\left(l_{i},t\right)\right)\left(L-2l_{i}-dl_{i}\right)dl_{i}$$

In order to calculate the thermal fields in the coil component cross section, the heat-conduction equation in cylindrical coordinates is given in Equation (5):

(5) $$\left\{ \begin{array}{l} \{\frac{\partial T(r,z,t)}{\partial t}=\frac{\lambda }{c\rho }\left(\frac{\partial^{2}T(r,z,t)}{\partial r^{2}}+\frac{1}{r}\frac{\partial T(r,z,t)}{\partial r}+\frac{\partial^{2}T(r,z,t)}{\partial z^{2}}\right)\\ \partial T{\left(r,z,t\right)}_{t=0}=T_{0}\\ \partial T{\left(r,z,t\right)}_{r=R_{Inner}}=T_{1}(t),\;\partial T{\left(r,z,t\right)}_{r=R_{Outer}}=T_{2}(t)\\ \partial T{\left(r,z,t\right)}_{z=Bottom}=T_{3}(t),\;\partial T{\left(r,z,t\right)}_{z=Upper}=T_{4}(t) \end{array}\right.$$

where *T*(*r,z,t*) is time-varying temperature field of the coil component material, *ρ* is density of the coil component material, λ is thermal conductivity of the coil component material, *c* is specific heat of the coil component material, *T*_0_ is initial temperature values of the coil component material, and *T*_1,2,3,4_(*t*) is the environmental temperature function in four boundaries of the coil component material. Using Equation (5), the numerical solution of the thermal field distribution in the cross section of fiber coil can be obtained by the finite element method (FEM). In other words, the thermal field distribution *T*(*r,z,t*) can be obtained with the ANSYS software, and the real-time temperature change rate of any loop fiber can be computed. Plugging these simulation data into Equation (4), the thermally induced rate error of the coils can be calculated.

## 3. Simulations and Experiments

### 3.1. Finite Element Model

**Figure 1:**
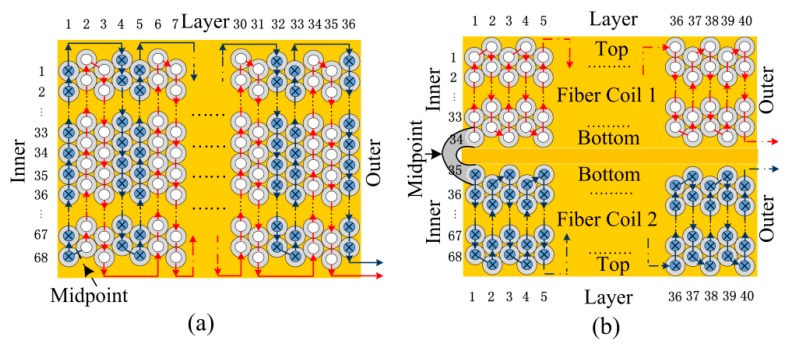
(**a**) QAD winding pattern; and (**b**) D-CYL winding pattern.

For investigating the temperature characteristics of IFOG with different winding methods, a classical QAD coil and a novel D-CYL coil are chosen. The winding techniques for the two winding methods are illustrated in Figure 1a,b, respectively. In Figure 1, the red arrow and blue arrow indicate the winding directions to each side of optical fiber, respectively. The yellow rectangle section, the bigger gray hollow circle section and the smaller filled circle section (white or blue) in Figure 1 represent glue material, coating and silica fiber core of the fiber coil, respectively.

The simulated geometric parameters of the two kinds of fiber coils are derived from our laboratory IFOG products. The geometric parameters of two kinds of fiber coils are almost the same, which can be designed as follows: The inner radius *R*_1_, outer radius *R*_2_, height *H*, layer number *M*, loop number *N*, and total loop number *MN* of the two kinds of fiber coils are 0.11 m, 0.121 m, 0.013 m, 40 layers, 68 turns, and 2720 turns, respectively. Additionally, the fiber length *L* and the effective diameter *D* of the two kinds of fiber coils are approximately 993 m and 0.115 m, respectively. Based on the geometric structure of the two kinds of fiber coils, the winding parameters of coils can be calculated, as illustrated in Table 1 and Table 2, respectively. 

The thermal parameters of the coils are provided in Table 3 [18,23,24,25].

It is clear from Figure 1a,b that the two winding methods have nearly same geometry parameters, however the winding methods show large differences. The D-CYL winding process is easier compared to the QAD winding method. The D-CYL winding method just needs to wind two small cylinder fiber coils in a specific direction, and then glue the two small coils together. However, the QAD winding method needs to wind one coil in two directions and the cross-layer winding tends to produce heavy winding flaws. Based on Table 1 and Table 2, we can calculate that the total fiber length of the D-CYL coil is 992.877508081 m, and that of the QAD coil is 992.877507888 m. The fiber lengths of both coils are almost the same, but the setting midpoint of the QAD coil is not the real fiber middle: the length from one end to the midpoint is 496.493638754 m, and the length from the other end to the midpoint is 496.383869134 m. Therefore, the QAD coil has no ability to provide spatial length symmetry on both sides of fiber coil midpoint, whereas the D-CYL coil happens to have the ability to maintain the greatest degree of symmetry of the spatial length of the fiber coil: the length from one end to the midpoint is 496.438754035 m, and the length from the other end to the midpoint is 496.438754046 m. Based on the foregoing analysis, we can hypothesize that a drift error may still exist in the QAD coil, even though a symmetrical temperature field is provided. It is also clear from Figure 1b that the D-CYL coil alone may not be capable of solving the drift error caused by axially asymmetrical temperature gradient fluctuations, while the QAD coil perhaps has limited capacity due to its winding structure characteristics shown in Figure 1a.

### 3.2. Simulation without Spool

**Figure 2:**
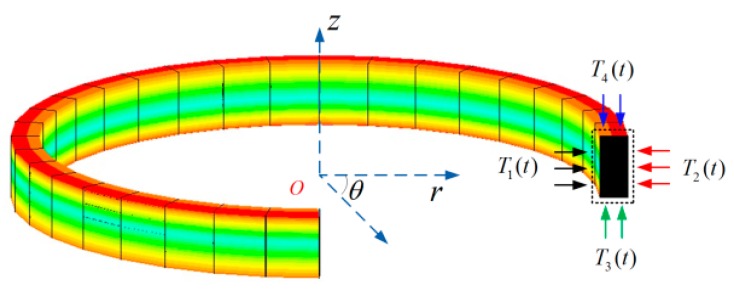
The frameless fiber coil and its cross section with indicated heat flow direction: $T_{3}(t)$ and $T_{4}(t)$ are axially heat flows, $T_{1}(t)$ and $T_{2}(t)$ are radial heat flows.

For verifying the correctness of the assumptions above, three comparative simulations of D-CYL and QAD coils without spool are performed. Here, we assume that the cross-section of the frameless coil is rectangular, as illustrated in Figure 2. 

**Figure 3:**
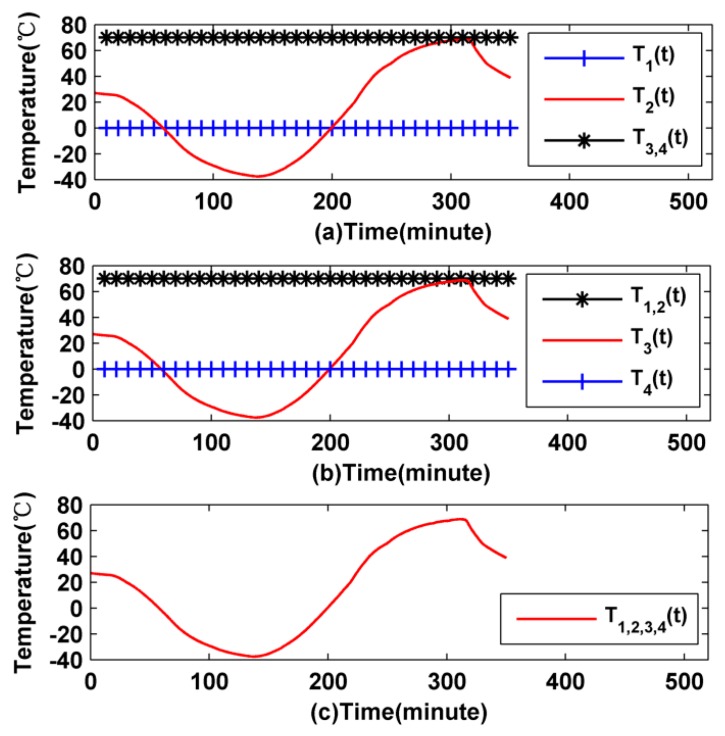
Simulation temperature conditions: (**a**) The radial asymmetrical temperature conditions; (**b**) the axially asymmetrical temperature conditions; and (**c**) the symmetrical temperature conditions.

In the first temperature scheme, the radial thermal excitation of fiber coils is asymmetric. The initial temperature *T*_0_ is set as 20 °C, the inner boundary temperature *T*_1_(*t*) is set to 0 °C, the outer boundary temperature *T*_2_(*t*) is changing over time, the upper boundary temperature *T*_4_(*t*) and the bottom boundary temperature *T*_3_(*t*) are all set to 70 °C, as illustrated in Figure 3a. In the second temperature scheme, the axial thermal excitation of fiber coils is asymmetric. The initial temperature *T*_0_ is set to 20 °C, the upper boundary temperature *T*_4_(*t*) is set to 0 °C, the bottom boundary temperature *T*_3_(*t*) is changing along with time, the inner boundary temperature *T*_1_(*t*) and the outer boundary temperature *T*_2_(*t*) are all set to 70 °C, as illustrated in Figure 3b. In the third temperature scheme, the initial temperature *T*_0_ is set to 20 °C, the four surface boundary temperatures *T*_1_(*t*), *T*_2_(*t*), *T*_3_(*t*) and *T*_4_(*t*) are all set as the same, as illustrated in Figure 3c. In the three temperature schemes above, the surface heat-transfer coefficient *h* is set as 5 W/(K·m), and each temperature excitation scheme lasts 350 min. 

**Figure 4:**
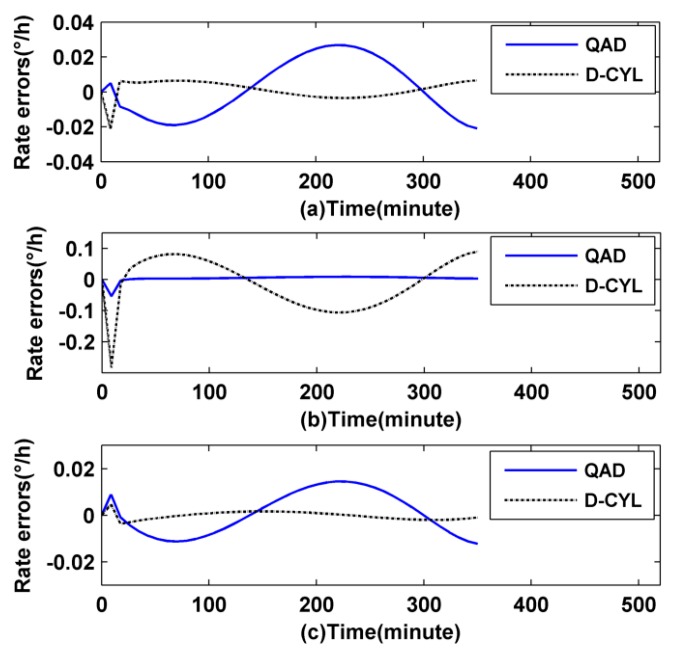
Rate errors of the IFOG without spool: (**a**) first temperature scheme; (**b**) second temperature scheme; and (**c**) third temperature scheme.

Based on the simulation values, the thermally induced rate error Ω*_E_*(*t*) of the two coils can be calculated as shown in Figure 4a–c, respectively.

It is clear from Figure 4b that the D-CYL coil itself has a poor thermal performance when it is provided with an axially asymmetrical temperature gradient fluctuation, while it does well with asymmetrical temperature fluctuation in the radial direction and symmetrical temperature fluctuation, as illustrated by a black dashed line in Figure 4a,c, respectively. The temperature performance of the QAD coil remains in a relative moderate range as shown by blue solid line in Figure 4. Such results reflect that, to a degree, the QAD coil has a certain adaptability when it faces a harsh temperature fluctuation, but the thermal performance of the QAD coil in response to symmetrical temperature fluctuations and radial direction asymmetrical temperature fluctuations is not good compared to the D-CYL coil, as illustrated in Figure 4a,c, respectively. As expected, the conclusions drawn from the simulation correspond to our prior hypotheses in this study.

### 3.3. Simulation with Spool

**Figure 5:**
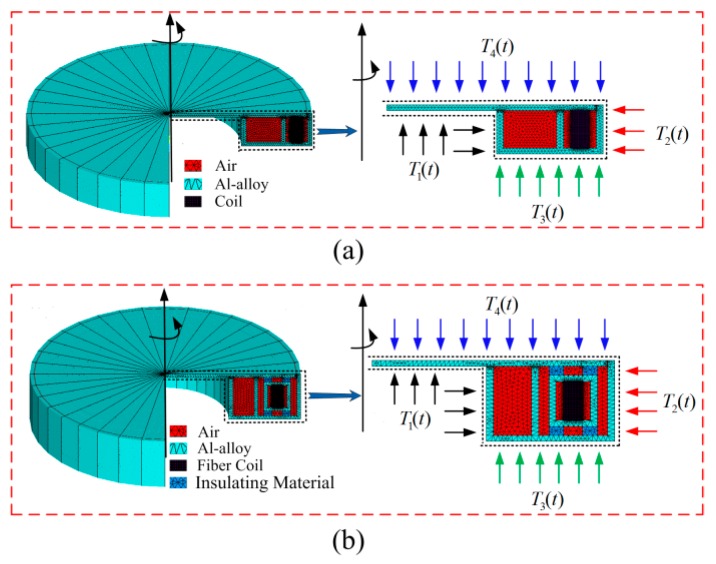
The fiber coils with its spools respectively and its cross section with indicated heat flow direction. *T*_3_(*t*) and *T*_4_(*t*) are axially heat flows, *T*_1_(*t*) and *T*_2_(*t*) are radial heat flows: (**a**) the old spool; and (**b**) the new design heat-off spool.

In the previous section, we have verified the performance of the QAD coil and D-CYL coil without spool in a harsh environment. Based on the simulation results, we find that the D-CYL coil itself really has poor ability when faced with an axially asymmetrical temperature fluctuation. To solve the problem, in this section we will show the performance of the QAD coil meshed with its old spool, the QAD coil meshed with its new spool, and D-CYL coil meshed with its new spool, respectively, under the same harsh temperature conditions. Figure 5a describes an old spool schematic diagram for a QAD coil, which was designed by our lab staff. Figure 5b describes a newly designed heat-off spool schematic diagram, which was recently designed by our research team [11]. 

**Figure 6:**
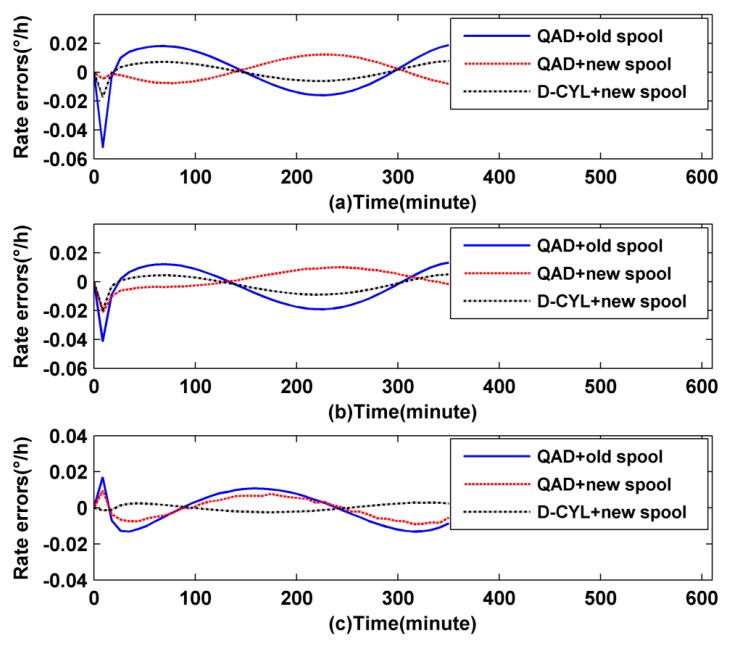
Rate errors of the IFOG with spool: (**a**) first temperature scheme; (**b**) second temperature scheme; and (**c**) third temperature scheme.

To comparatively analyze the temperature performance of the three kinds of combination (the first combination refers to the QAD winding coil meshed with the old spool, the second refers to the QAD coil meshed with the new heat-off spool, and the third refers to the D-CYL coil meshed with the new heat-off spool, respectively), three temperature schemes are adopted. The simulation temperature conditions are the same as the conditions in the previous section, as illustrated in Figure 3a–c, respectively. The homologous thermally induced rate error Ω*_E_*(*t*) of the three kinds of combination can be calculated as shown in Figure 6a–c, respectively.

It is clear from Figure 6a–c that once the D-CYL coil is subjected to axially symmetrical temperature conditions, it will display better performance in suppressing phase shift errors than the QAD coil. Therefore we can draw the conclusion that a D-CYL coil meshed with a heat-off spool can overcome the axially asymmetrical temperature fluctuations. Meanwhile we also find that the thermal performance of a QAD coil with its old spool is worse than that without spool under certain temperature conditions, as shown in Figure 4a,c and Figure 6a,c, respectively, but this situation will be somewhat improved when the QAD coil is meshed with a new heat-off spool.

The reason maybe is that the old spool (Figure 5a) is not perfect while the new heat-off spool (Figure 5b) is relatively perfect. As we all know, fiber coils have to be meshed with a kind of metallic spool to protect them in practical application, therefore the old spool does not act completely as a temperature homogenizer in its original design but was used to protect the fiber coil. However the new heat-off spool does not act merely as a protective cover for the QAD coil, but it can also act as a temperature buffer homogenizer. It is also clear from Figure 6 that with the help of the heat-off spool the D-CYL coil has the best temperature performance compared to the other two coil combinations, even though the outer temperature conditions are asymmetrical. Based on the simulation analysis above, we can conclude that the thermal performance of a D-CYL coil meshed with the new spool is better than that of a QAD coil meshed with an old spool or a new spool under harsh temperature conditions.

### 3.4. Experiment Section

In order to make the simulation results more persuasive, two experiments were performed. To ensure experimental accuracy, the IFOGs are divided into two parts. The three fiber coil combinations with their homologous spools (QAD coil meshed with old spool, QAD coil meshed with heat-off spool and D-CYL coil meshed with heat-off spool) are put into a temperature test chamber, and the other parts are put into the room environment. 

**Figure 7:**
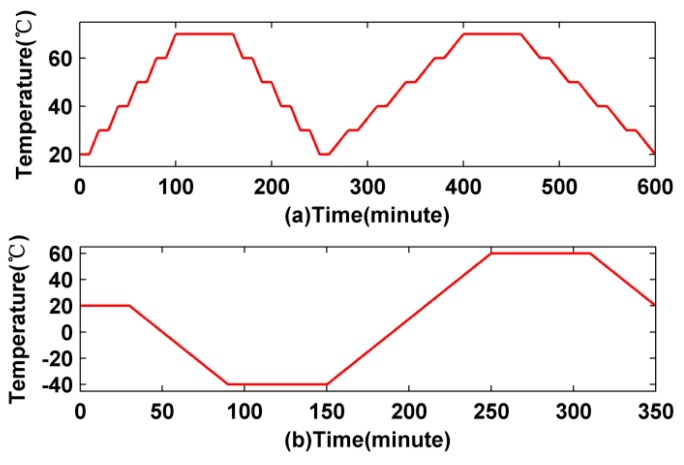
Experiment temperature load scheme: (**a**) the first load scheme; and (**b**) the second load scheme.

In the first experiment, the temperature is held for 1 h at a temperature point of 70 °C and then held for 10 min at temperature points of 20, 30, 40, 50, and 60 °C, respectively, as shown in the red line of Figure 7a. To make the experiments more comprehensive, two different temperature change rates including 1 °C /min and 0.5 °C /min are also used. In the second experiment, the temperature is held for 30 min at a temperature point of 20 °C and then held for 1 h at temperature points of −40 °C and 60 °C, respectively, as shown by the red line of Figure 7b.

**Figure 8:**
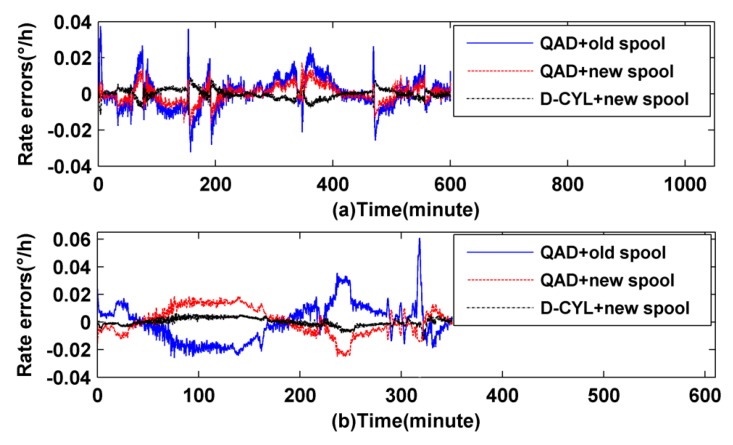
Rate errors of IFOG in experiments: (**a**) the first experiment and (**b**) the second experiment.

To eliminate the noise influence, the output signal is processed by subtracting the mean values of the original signals as shown in Figure 8. The blue solid line, red dotted line, and black dashed line in Figure 8 indicate the IFOG experimental values with the first, the second, and the third combination, respectively. The experimental results show that the IFOG with a D-CYL coil meshed with a new heat-off spool has higher accuracy than that with QAD coil meshed with an old spool or new heat-off spool, which essentially verifies the simulation results. 

## 4. Analysis and Discussion

It can be seen clearly from Figure 6a–c that the D-CYL coil meshed with a heat-off spool has better performance than the QAD coil meshed with an old spool or a new design heat-off spool. The reason is that the D-CYL coil not only has a spatially symmetric distribution on both sides of the fiber coil midpoint, but also it can be provided with an axially symmetric temperature by the heat-off spool, therefore the thermally induced drift error could be essentially counteracted. We also discover from Figure 6 that the performance of a QAD coil meshed with its heat spool is at the middle level compared with the other two forms. The reason may be that the QAD coil does not have a spatially symmetric distribution on both sides of the fiber coil midpoint, even though it is provided with an axially symmetric temperature by its heat-spool. The two points above further verify the importance of the combination of both a spatial length symmetric distribution and a symmetric temperature distribution of the fiber coil. Then we can boldly conclude that the performance of the QAD coil meshed with the old spool is the worst of the three simulation groups. The simulation results just confirm this corollary, as shown in Figure 6. Besides, the simulation results from Figure 4b also reflect the fact that the D-CYL coil itself has little capacity to suppress the axially temperature gradient compared to a QAD winding coil, while once the coil is provided with a heat-off spool, the fragile axially thermal performance could be improved, as illustrated by the black dashed line in Figure 6b. The grounds for this include the following: the outer shield of the heat-off spool is used to equalize the surface temperature by smoothing out the local heat source fluctuations, which breaks the asymmetry of heat flows. The nearby layer is air, which can prevent the thermal waves from penetrating into the coil, thus reducing the rate of temperature change throughout the coil volume. The inner layer is used to equalize the temperature throughout the coil surface by symmetrizing the penetrating heat flows, similar to the outer shield. Therefore we can draw a conclusion that the newly designed heat-off spool could suppress the axially asymmetrical thermal fluctuations for a D-CYL coil. Meanwhile we also find that the drift error of a QAD coil cannot be nicely counteracted even though it is provided with a symmetrical temperature environment as shown by the blue solid line in Figure 4c and red dotted line in Figure 6c. Considering the characteristic winding of the QAD winding method, we find that there are two profound reasons. One reason is the heat-transfer time-delay of nearby layers of fiber coil. The other reason is that the setting midpoint of the QAD fiber is not the real fiber middle, so a drift error always exists. 

The simulation results above verify the hypothesis, to a certain degree, only under ideal conditions, but in fact, the fiber coils and spools are destined to have production process defects. The defects are caused by several factors: fiber drawing defects, winding defects, metal structure shape changes and so on. For further verifying the correctness of the hypothetical results, two comparative experiments were implemented as shown in Figure 8a,b, respectively. It is clear to see from the red dotted line in Figure 8 that with the help of a heat-off spool, the QAD IFOG’s maximum thermally induced bias value can be minimized to 0.03°/h. If the QAD winding method is meshed with an old spool, the maximum thermally induced bias value will increase to 0.06°/h, as shown by the blue solid line in Figure 8. Through careful observation of the red dotted line and blue solid line in Figure 8 we also find that the heat-off can effectively alleviate any sharp fluctuations for a QAD IFOG, especially at the start, and during heating and cooling time. Simultaneously, regardless of whether the QAD coil is meshed with a new heat-off spool or an old spool, its thermally induced curve levels off under low temperature conditions, as shown in Figure 8b. The reason may be that the fiber and adhesive are more sensitive to high temperatures than low temperatures. Compared to the two experimental schemes above, the D-CYL scheme has a better temperature performance. Its maximum thermally induced is only 0.012°/h. During the start, heating or cooling time, the thermally induced drift error can be effectively attenuated. Such good results may be attributed to a perfect combination of the heat-off spool and D-CYL winding method, which can provide fine performance.

According to the comparison and analysis above, we can draw a conclusion that the QAD coil itself could suppress the asymmetrical thermal fluctuation influence to a certain extent and the thermal performance can be improved slightly with the help of a temperature homogenizer. However, only with the aid of a heat-off spool is an axially symmetrical temperature environment provided, and the D-CYL coil can achieve fully its thermal performance. Through the analysis and discussion mentioned above, we can now say that a D-CYL winding coil meshed with a heat-off spool has a better thermal performance than the traditional QAD products. The results fully verify the superiority of the D-CYL method in suppressing thermally induced bias drift errors.

## 5. Conclusions

The thermal performance of fiber coils wound by the QAD and D-CYL methods with the same fiber length are comparatively analyzed in this article, and the advantages of the D-CYL fiber coil have been demonstrated by simulation and experimental results. Besides, under the condition of an axially asymmetrical temperature load, the rate error of a D-CYL coil is larger than that of a QAD coil, but the weakness of the D-CYL fiber coil in axial direction can be overcome by adding a heat-off spool. With the aid of this heat-off spool, the QAD IFOG’s thermally induced bias error can also be decreased slightly. To further improve the thermal performance of the D-CYL fiber coil, future efforts will focus on the research of new graphite adhesive and spray adhesive technology, and D-CYL fiber coils and cross fiber coils with double skeletons will also be considered in our future work.

## Figures and Tables

**Table 1:** Winding parameters for the D-CYL fiber coil.

*i*	*M*	*N*	*l_i_/m*	*dl_i_/m*
1	40	1	0	0.379652746 m
2	40	2	0.379652746 m	0.379652746 m
⋮	⋮	⋮	⋮	⋮
33	40	33	12.148887872 m	0.379652746 m
34	40	34	12.528540618 m	0.379652746 m
⋮	⋮	⋮	⋮	⋮
1360	1	34	496.09269035 m	0.346055 m
1361	1	35	496.438754035 m	0.346055 m
⋮	⋮	⋮	⋮	⋮
2687	40	35	979.969314717 m	0.379652746 m
2688	40	36	980.348967473 m	0.379652746 m
⋮	⋮	⋮	⋮	⋮
2719	40	67	992.118202589 m	0.379652746 m
2720	40	68	992.497855335 m	0.379652746 m

**Table 2:** Winding parameters for the QAD fiber coil.

*i*	*M*	*N*	*l_i_/m*	*dl_i_/m*
1	40	68	0	0.379652746 m
2	40	67	0.379652746 m	0.379652746 m
⋮	⋮	⋮	⋮	⋮
67	40	2	25.057081236 m	0.379652746 m
68	40	1	25.436733982 m	0.379652746 m
⋮	⋮	⋮	⋮	⋮
1360	1	68	496.147583754 m	0.346055 m
1361	2	68	496.493638754 m	0.347168 m
⋮	⋮	⋮	⋮	⋮
2653	39	1	967.126383888 m	0.378693 m
2654	39	2	967.505076888 m	0.378693 m
⋮	⋮	⋮	⋮	⋮
2719	39	67	992.120121888 m	0.378693 m
2720	39	68	992.498814888 m	0.378693 m

**Table 3:** Parameters for calculation.

Parameters	Al-Alloy	Core	Coating	Glue	Insulating Material
Density *ρ* kg/m^3^	2740	2203	1190	970	2520
Specific heat *c* J/(kg·K)	896	703	1400	1600	2000
Thermal conductivity λ W/(K·m)	221	1.38	0.21	0.21	1.6
